# Grief and the non-death losses of Covid-19

**DOI:** 10.1007/s11097-022-09878-8

**Published:** 2022-12-21

**Authors:** Louise Richardson, Becky Millar

**Affiliations:** Department of Philosophy, University of York, YO10 5DD York, UK

**Keywords:** Grief, Loss, Covid-19

## Abstract

Articles in the popular media and testimonies collected in empirical work suggest that many people who have not been bereaved have nevertheless grieved over pandemic-related losses of various kinds. There is a philosophical question about whether *any* experience of a non-death loss ought to count as grief, hinging upon how the object of grief is construed. However, even if one accepts that certain significant non-death losses are possible targets of grief, many reported cases of putative pandemic-related grief may appear less plausible. For instance, it might be argued that many of these losses are temporary or minor and therefore unlikely to be grieved, and that the associated experiences are phenomenologically dissimilar to grief. In this article, as well as discussing the more general question about the coherence of the idea of non-bereavement grief, we address these obstacles to taking reports of pandemic non-bereavement grief to be literal and true. In particular, we argue that some may have experienced grief over even apparently minor losses during the pandemic. This is generally so, we suggest, only insofar as experiences of such losses form part of an overarching *grief process* directed at some broader significant loss. Thus, we cast light on both the nature of non-bereavement grief and the kinds of disruption and loss experienced during the pandemic.

## Introduction

The Covid-19 pandemic has made grief salient for a number of reasons. Most obviously, as our attention has been drawn to deaths caused by the SARS-CoV-2 virus, so we have also been made aware of grief over those deaths. Furthermore, the experience of those bereaved during the pandemic–whether due to Covid-19 or not–has been affected in many countries by restrictions intended to reduce the spread of the disease or protect those most vulnerable to it (see Ratcliffe, this issue). In addition, articles in the popular media and testimonies collected in empirical work suggest that many people who have not been bereaved have nevertheless grieved over pandemic-related losses of various kinds. These purported non-bereavement losses of the pandemic will be our focus here. There is a philosophical question about whether *any* experience of a non-bereavement loss ought to count as grief, hinging upon how the object of grief is construed. However, even if one accepts that certain significant non-death losses—of one’s home or one’s job, for example—are possible targets of grief, numerous other reported cases of putative pandemic-related grief may appear less plausible. For example, it might be argued that such cases are insufficiently phenomenologically similar to bereavement grief, or that they can be discounted on the grounds that the losses incurred are impermanent or too minor to be grieved. If these objections are upheld then we might conclude that talk of pandemic non-bereavement grief is either mistaken, not to be taken literally, or that it uses ‘grief’ in a different sense to that employed to refer to our emotional response to bereavement.

This is how our discussion proceeds. In Section 2 we set out the types of non-bereavement losses that have been reported during the pandemic. To make conceptual space for such reports to be both literal and true there is a philosophical issue to be addressed about the object of grief. In Section 3 we discuss this general question, noting that few philosophers have sought to rule out non-bereavement grief, and that one account in particular explicitly accommodates non-bereavement grief by taking its object to be a loss of significant *life possibilities*. However, even if we accept such an account of grief’s object, there are obstacles to the idea of grief over the non-death losses *of the pandemic*, specifically. In Sections 4 and 5 we argue that these obstacles can be overcome.

## ‘All the things we have to mourn now’^1^

Early in the Covid-19 pandemic, popular articles suggested that some of the emotional upheaval that people were feeling was grief. Some of us, it was proposed, even if we had not been bereaved, were grieving losses incurred due to the arrival of Covid-19 or the restrictions introduced in response to it. These articles typically cited or were written by grief experts. For instance, David Kessler, originator with Elisabeth Kübler-Ross of the idea of five stages of grieving, when asked whether some of what people were feeling was grief answered ‘yes, and we’re feeling a number of different griefs’, over, for example, loss of normalcy, loss of connection, and loss of safety (Berinato, 2020). In an interview for *The Atlantic*, Pauline Boss–known for her work on ambiguous loss–suggested that we might grieve for ‘major losses’ that ‘have to do with the relationship between ourselves and the changing world’: ‘the loss of a routine, the loss of freedom to go out and do what we please, the loss of being able to hug our loved ones and be with our friends.’ On Boss’s view, we need to name these losses, and she is explicit in describing what we feel in response to them ‘grief’ (Pinsker, 2020). Psychotherapist Lori Gottlieb, writing in the *New York Times*, singled out grief over specific and ‘smaller’ losses, such as cancelled celebrations, sporting events and holidays, as well as pointing to an experience of a more diffuse loss of ‘predictability’ due to our suddenly finding ourselves unable to do much of what we previously took for granted. Like Boss, Gottlieb emphasised the need, as she saw it, to acknowledge this grief (Gottlieb, 2020). Much later in the pandemic, the idea that it had brought grief even to the non-bereaved still resonated. For example, a short film that appeared on the *New York Times* website in December 2021 titled ‘Grieving our old normal’ suggested that, as the pandemic progressed, it felt for some of us that ‘the timeline we were on, was suddenly gone’, and with it ‘our old selves’, for whom we might feel grief. This, it was suggested, might involve experiencing not only the loss of things that one had confidently expected, such as a wedding or holiday, but also losses related to things one had never and now would never have such as a potential partner we never met (Crouse et al., 2021).

Some empirical work has also generated descriptions of what might be understood as non-bereavement grief during the pandemic. For instance, the Pandemic Experience Corpus includes many hundreds of responses to the question ‘Have you felt any sense of grief or loss over other aspects of life that have changed because of the pandemic? If so, what has affected you most?’ (Froese et al., 2021).^2^ Those who responded to this question in the affirmative reported feeling grief or loss over, for instance, cancelled events (e.g., weddings, holidays, graduations); prohibited activities (e.g., shopping, attending church, swimming, hugging, visiting family); and departed presuppositions and attitudes (‘how I imagined my life would be’, ‘my trust in other people’). Descriptions of grief over non-death losses cut across divisions in attitudes towards pandemic restrictions. For instance, some reported feeling a loss of liberty, whilst others described grief or loss associated with how they believed the pandemic had been handled or how others had responded: ‘I grieve for the complete fuck-up my country is’; ‘the loss of tolerance, compassion and understanding in some people towards people who are at high risk’. There are also respondents whose sense of loss relates to the ending of restrictions: ‘the strongest sense of loss has been coming out of the calm and quiet of lock down back into the madness of normality.’ In another study—the COVID-19 Coping Study—older adults (55+) were asked whether there was anything they were ‘grieving, mourning the loss of, or sad about.’^3^ Respondents identified a wide range of felt losses, many overlapping with those described above, such as grief associated with cancelled events and the absence of physical intimacy, as well as more overarching losses of, for example, ‘what was normal everyday life’ or ‘the illusion of control’.

## The object of grief

While the idea of grieving over losses such as those enumerated above seems to come quite naturally to us, it is nevertheless an open question how such talk should be understood. Not all uses of ‘grief’ aim to pick out emotional responses to loss: consider ‘he was giving me grief over the money I owed him’. In addition, even if we take some descriptions of non-bereavement grief literally, we might still ask whether what is felt in such cases is the grief one might feel following bereavement, or some other emotional response to loss or change different in kind from grief. This possibility is made salient by the fact that the survey studies mentioned above ask participants about their sense of ‘grief or loss’, rather than grief specifically.^4^ In addressing such issues it should bear some weight that theorists and clinicians already use ‘grief’ to describe the emotional experience of loss other than bereavement (e.g., Harris, 2020). And, some empirical studies support the idea that there is overlap between the experience of bereavement and of a variety of non-death losses such as for example, involuntary rehousing (Archer & Hawes, 1998), job losses and divorce (Papa et al., 2014), as well as non-death losses associated with natural disaster (Shear et al., 2011). Nevertheless, one might think that there is further and distinctively philosophical work to be done to establish the limits of grief—whether it can take non-death losses as its object, and if so, which ones. For instance, if it were the case that experiencing grief necessarily involved representing that some individual S has died, then grief over non-death losses would be ruled out. If this were correct, we could accommodate the already-accepted use of ‘grief’ to describe non-death losses by taking it to be non-literal, such that the different uses of the term pick out two different kinds of experience. Strictly speaking, on this view, there would be grief (in the bereavement case), and a distinct kind of loss experience.

In assessing this suggestion, it is helpful to note that extant philosophical work on grief does not typically rule out all non-death grief experience, though it often sets it aside and rarely says much about it.^5^ For instance, Daniel Gustafson argues that grief is subject to a ‘belief condition’ that is in paradigm cases satisfied by the grieving subject’s possession of a belief ‘to the effect that a certain person is dead, has died, or has been almost certainly lost or permanently separated’ from the grieving subject (1989, p. 464). This allows for grief over *personal* losses (i.e., losses of persons) other than bereavements, and Gustafson also entertains though does not clearly endorse—he suggests we can ‘almost imagine’—the idea that the belief condition could be met in the case of the loss of a ‘nonliving entity’. Similarly, Michael Cholbi argues that ‘grief’s formal object is the transformation of the bereaved’s relationship with the deceased’ (2021, p. 60). Nevertheless, whilst he suggests that bereavement grief is ‘likely to be our paradigm case’ of grief, he allows that much of his account ‘is consistent with, and applicable to, other ‘griefs’, or grief-like states besides the mental states prompted by another’s death’ (p. 190). For instance, he emphasises that we grieve those in whom we have invested our practical identities, and this is something that could plausibly be adapted to losses of anything (e.g., careers, homes, hobbies) playing an identity-conferring role.^6^ Whilst Robert Solomon focusses wholly on bereavement grief, he observes that a limit of his discussion of grief is that he is concerned with a single ‘paradigm’ of grief, and allows that ‘there are others’. Furthermore, his paradigm is not even bereavement grief but, more narrowly still, the grief that follows the ‘more or less sudden loss of a loved one’ (2004, p. 77). Likewise, though Berislav Marušić describes ‘her being dead’ as the primary object of his grief over the death of his mother, he also mentions, in passing, ‘grief over a breakup’ (2018, p. 6). Janet McCracken rules out some non-death loss experience as grief, since it will not involve experiencing the ‘obligatory, dedicatory qualities’ that she takes to be a crucial aspect of grief (2005, p. 142). However, she stops short of denying that there can be non-bereavement grief, observing that ‘it may make sense to say that one feels real grief over the devastation of a great building by war, or the destruction of one’s house in a natural disaster’ (p. 143).

There is then no argument against non-bereavement grief to be found in these philosophers’ accounts: on the contrary, they want to allow for it. Neither though do they substantiate the idea of non-bereavement grief by explaining what the object of grief could be such that both bereavements and other losses can be grieved for. By ‘object of grief’ we mean both what in the world grief is about when it is appropriate or fitting, but also what a grief experience *seems* to be about.^7^ The idea that grief’s object makes room for non-bereavement grief can be substantiated. For instance, partly in order to accommodate some forms of non-bereavement grief, Somogy Varga and Shaun Gallagher propose that ‘responding to a loss of opportunities’ is a necessary feature of grief (2020, p. 177). This allows for grief over a range of non-personal, non-death losses, as well cases of *personal* loss in which the loss of opportunities is merely anticipated, wholly vicarious or ‘anticipatory vicarious’, as when one grieves for the lost opportunities that someone else will incur when one dies.

More explicitly addressed at the question of grief’s object, but seemingly consistent with Varga and Gallagher’s proposal, Matthew Ratcliffe argues that the primary object of grief is a loss of *life possibilities*: ‘significant possibilities that are integral to the structure of one’s life, to one’s various projects, pastimes, habitual activities, and commitments’ (Ratcliffe et al., 2022, p. 12).^8^ In bereavement grief, these life possibilities are ones that depended on the continued presence of the deceased. They can be the possibilities of the bereaved, the deceased, or shared possibilities. Thus, even in this putative paradigm case, grief is not ‘principally about the subtraction of something concrete from one’s world’ (ibid.). Hence, this account extends the bounds of grief, in principle at least, to cases that involve the loss of no concrete object. For example, more diffuse experiences of grief that might be described as relating to a loss of safety or trust in others might each be understood as the experience of the loss of a network of significant life possibilities. Likewise, the view allows for grief over that which one never had, such as a partner you did not meet or a child who was never born or conceived: ‘objects’ (in a loose sense) compounded of future possibilities. This approach thereby fills in important details left out by other accounts, which do not wish to rule out non-bereavement grief, but fail to explicitly substantiate the idea.

Though we take it to be phenomenologically plausible, we do not seek to defend the lost possibilities account of the object of grief here. It may be possible to formulate alternative philosophical views of grief’s object that, like this account, substantiate the idea of non-bereavement grief. Indeed, insofar as continued life possibilities are central one’s confidence in the world, the lost possibilities account can be thought of as a more precise formulation of a widely accepted view of grief according to which it crucially involves engaging with profound disturbance to what Colin Parkes labelled the ‘assumptive world’ (Parkes, 1988).^9^ What is most important for our purposes is that if one accepts such an account, there is conceptual space for grief over non-death losses. However, this does not settle the question of whether the specific pandemic loss experiences we are considering ought to be construed as grief. Therefore, rather than pursue the general question of non-bereavement grief further, we will in the rest of this paper consider the narrower question of whether reports of non-bereavement grief *during the pandemic* can be understood as both literal and true. Where the discussion proceeds in terms of lost possibilities, we believe that it could be reformulated in terms of some other suitably expansive account of grief’s object, if one is forthcoming.

Before we move on to pandemic non-bereavement grief, note that endorsing an expansive account of grief’s object–and thus its bounds–does not entail denying that there might be important features typical to certain kinds of grief. Bereavement, and other personal losses, such as those we might experience when a person goes missing or is in a persistent vegetative state, may involve interpersonal features that will be absent from other kinds of loss experience. For instance, the ‘dedicatory’ quality mentioned by McCracken may be one such. It is consistent with this that there is a kind of experience that it is appropriate to call ‘grief’, and which is not restricted to personal loss, still less to bereavement.

## Two obstacles: impermanence and phenomenological dissimilarity

Given the discussion of the previous section, we can allow that there might be grief over the loss of, for example, a home, a job or a marriage, losses not infrequently incurred both during and outside of the pandemic. These are all things upon which very significant life possibilities (and more generally, an assumptive world) may and often do depend. Setting such cases aside though, there are reasons to be cautious over many of the losses that were described as grieved over during the pandemic. In the next section we consider the charge that many of these are too minor to be grieved for. In this section we address two issues that would arise even for pandemic losses agreed to be more significant than venue closures and event cancellations: first, that they are obviously impermanent and so unsuited for grief, and second, that the experience of such losses is phenomenologically dissimilar to grief.

To begin with, it seems that the grief felt over the death of a person cannot be divorced from the fact that such a loss is permanent and more importantly known to be permanent. It might seem therefore to be a condition on counting any loss experience as genuine grief that the relevant loss is likewise understood by the subject as enduring. However, many of the non-death losses of the pandemic were temporary: most people have been able to meet up with loved ones again and return to work and other activities. Given the temporary nature of these losses, one might think, they cannot have been objects of genuine grief. Appeal to the category of ambiguous loss might go some way to assuaging this worry. Ambiguous losses are ones that ‘remain vague and uncertain’ (Boss, 1999, p. 5), the uncertainty being part of what sometimes makes such losses so distressing and hard to ‘move on’ from. An ambiguous loss would be suffered by, for example, the family of a missing soldier, when it is not known whether they will return and thus whether the loss is permanent or not. However, though this may allow us to call ‘grief’ what is felt over a loss that is not unequivocally understood by the subject to be permanent, still, one might think, we should resist the idea of grief over losses that are understood to be *non*-permanent, as must be the case with some of what was lost during the pandemic. As one contributor to the Pandemic Experience Corpus put it in response to the question of whether they felt a sense of grief or loss, ‘not really, because I expect that in time all the things currently closed to me will become available again.’

Other testimonies, however, suggest a different picture. For example, some (especially the older respondents in the COVID-19 Coping study) describe feeling that they had little time left:

‘I realize that as an older person (74) there are things I should not resume. I am aware that I don’t have many years left, and this is robbing me of time’, shared Barbara. ‘At my age, valuable time is running out’, wrote Debra (76 years). Karen (88 years) grieved ‘missing out on what life I have left’. (Statz et al. 2022, p. 4)

Losses that we might be tempted to think of as temporary, when reported in the context of this kind of feeling of restricted time, are plausibly understood by the subject either as permanent (‘I have little time left and so will not be able to do x again’) or at least not as non-permanent (‘I have little time left and so may not be able to do x again’). This was made explicit by some subjects:

As an older person the loss of time affects me most, and the possibility that opportunities may have been lost and it will not be possible to reinstate them.

Factors other than the feeling that one’s own time is short also affect whether a subject understands a loss to be temporary or instead permanent or not non-permanent. For instance, in the Pandemic Experience Corpus, losses were also reported in the context of feeling that things would not or might not return to how they were before, or because the window of time within which it was possible for something to happen, had passed:

I feel a sense of loss over the sports and social activities that I used to do, because I’m not sure that I will ever do some of them again.

I have felt a small sense of loss of youth, as often people say your twenties is a time of great adventure, which has been taken from me and many others. And time is not something that can be given back.

Hence, ruling out grief over non-death losses during the pandemic on the grounds of non-permanence would be a mistake. No doubt some losses were understood to be temporary and so will not have been grieved for. However, there is evidence that some subjects either understood their losses to be permanent, or as not non-permanent, in which case their losses can be considered ambiguous ones. In appealing to future losses (their own death for example, or current losses persisting due to things not returning to normal) these subjects’ grief may also be considered in part–although not wholly–anticipatory.^10^

The second potential obstacle to thinking that—beyond obvious cases such as job-losses—the experiential disruption reported during the pandemic was non-bereavement grief is, simply put, that what people felt during the pandemic just did not feel as grief feels. We suggest, in contrast, that there is substantial phenomenological overlap between the two.

Grief’s phenomenology is typically taken to centrally involve wide-ranging disruption to experiences of both the world and of one’s own bodily and affective states. As noted above, bereavement is often taken to involve a shattering of one’s ‘assumptive world’ – the core system of beliefs that orient us in the world (Parkes, 1988). Consistent with this idea, philosophical accounts of grief emphasise dramatic disturbances to one’s experiential world and the breakdown of abilities to negotiate this world (e.g., Maclaren, 2011; Ratcliffe, 2017; Millar, 2021), as well as the process of ‘relearning one’s world’ in response to such disruption (Attig, 2011). In line with these depictions of grief’s phenomenology, the pandemic has disrupted many people’s experiences. According to researchers, events of the pandemic may have shattered some people’s assumptive worlds (e.g., de Jong et al., 2020; Richardson et al., 2021), compromising, for example, core beliefs regarding safety and stability. Similarly, amid the restrictions and new norms of the pandemic, one may have had to navigate a new and unfamiliar world, necessitating a gradual process of ‘relearning’ and adaptation.

Grief also often involves unsettling bodily and affective dysregulation. This hinges in part upon changes to the affective scaffolding and regulatory resources available. Our bodies and emotional experiences are regulated in many important ways by aspects of the surrounding world, activities, practices, and other people (e.g., see Colombetti & Krueger, 2015; Krueger, 2014)—something that especially comes to the fore in times of stress or upheaval. For example, when faced with a threat, holding one’s partner’s hand can attenuate the activation of the neural systems involved in threat responses (Coan et al., 2006), and this kind of social touch can even buffer experiences of physical pain (e.g., von Mohr et al., 2018). Bereavement often involves the unfortunate irony of losing the very person who would have helped one to negotiate the disruption of grief (e.g., see Ratcliffe & Byrne, 2022; Ratcliffe, 2022, Chap. 7), making the experience even more distressing.^11^ For many, the pandemic—much like bereavement—involved dramatic alterations to available sources of affective scaffolding, most obviously through lockdowns and other social restrictions. During the initial lockdown in the UK, face-to-face contact with people outside of one’s immediate household was banned, and ‘non-essential’ businesses and activities were shut down. Many people were deprived of physical contact with others, with some experiencing weeks or months of isolation and ‘touch hunger’ (e.g., Durkin et al., 2020).^12^ We might therefore expect comparable emotional and bodily disruption in bereavement and pandemic-related losses due to their analogous capacities to profoundly impact available social support.

The social restrictions of the pandemic clearly limited or ruled out activities and hobbies that involve other people, such as team sports and club nights. However, various solitary activities and practices that would ordinarily serve to scaffold one’s affective experiences were affected too. For instance, during the initial lockdown in the UK, government guidance limited outdoor exercise alone to once per day. Even where activities were within the rules, societal or moral pressure to stay at home may have made them less appealing and apt for emotion regulation. Although for different reasons, bereavement can likewise involve important alterations to the activities and pastimes available as regulatory resources. In particular, many activities may *implicate* the person who died, and therefore no longer be desirable or even intelligible, to the extent that they rely upon that person (e.g., Maclaren, 2011; Ratcliffe, 2019). As Ratcliffe (2019) puts it, certain activities may be *for* that person, or they might relate to things done together *with* that person, and the ‘we’ involved in such activities may not be reducible to a ‘you’ and an ‘I’. Perhaps walking in the park or sitting down for a meal had been an important means of improving one’s mood, but had always been shared with the person who died. As Maclaren puts it, ‘without her there to do her part in the dance that awakened those meanings, carved out those routes, and reminded him of his own place, now even breakfast seems unmanageable’ (2011, p. 60). Rather than affording opportunities to beneficially regulate one’s mood, such activities may cease to make sense to the bereaved or serve as overwhelming reminders of the absence of their loved one. Thus, due to both bereavement and pandemic loss experiences holding the potential for significant worldly disruption and changes to available sociocultural affective scaffolding, we ought to expect some important phenomenological overlaps between the two. This is not to deny, however, that grief experienced in different circumstances might also have characteristic phenomenological *dissimilarities*. At the end of Section 3 we noted that grief over personal losses might have some distinctive features. The phenomenology of much pandemic-related non-bereavement grief might for example be affected by the fact that (unlike in the case of bereavement) the losses incurred were widely shared.^13^

## Cancellations and closures: grief over ‘minor’ losses

Although *prima facie* dissimilarities with bereavement grief in terms of permanence and phenomenology fail to rule out grief over pandemic losses, another challenge arises. Many of the non-bereavement losses during the pandemic that have been described as grieved for in the popular media seem not to involve the wide-ranging, world-shattering losses that are characteristic of paradigmatic grief. What are we to make of cases like alleged ‘grief’ over the cancellation of events, or closures of sporting or entertainment venues? Given that grief is typically characterised by profound disruption, suffering, and emotional upheaval, one might worry that allowing for grief over these seemingly minor losses *trivialises* the experience. People’s feelings in relation to cancellations and closures may be better described as, for example, sadness or disappointment. Whether we allow for grief over losses like cancellations and closures is not merely taxonomical. Downplaying, or failing to recognise, the extent of suffering involved in grief is plausibly morally and not just conceptually dubious. In this section, we respond to this worry and argue that even these seemingly minor losses of the pandemic can be genuine objects of grief.

As a first line of response, it is important to note that what counts as a *minor* loss varies among individuals, depending upon their interests, values, and the overall structure of their life. For some people, something such as a wedding or school prom might take on such significance that they experience profound and wide-ranging loss and disruption when the event is cancelled; such an event could in principle become central to their assumptive world. Moreover, as discussed in the previous section, during the pandemic such losses occurred against a backdrop of diminished affective scaffolding, and so losses that one would ordinarily be able to cope with may have taken on a heightened significance. For example, without having other people to provide comfort or other activities to distract oneself with, a cancelled much-anticipated event might itself be experienced as a major loss. Cancellations and closures might thereby be themselves elevated to significant losses, of the sort that more ordinarily trigger grief.

To allow this is not to trivialise grief. When it occurs in the context just envisaged, the cancellation of the event (say) would be a significant source of suffering. At this point, those worried about trivialisation might retort that even if such losses *are* as a matter of fact sometimes significant enough to be met with grief, they *should not be*. Events and activities of the kind that were cancelled or prohibited during the pandemic, so goes the objection, should not take on such significance in a life that their loss occasions grief. To this, we can respond that this is not our concern here: we are addressing the descriptive question of whether certain experiences are grief or not, not the normative question of when what is in fact grief ought and ought not to be felt. We can allow that an experience of grief may be appropriate in that it accurately represents a loss for its subject but still be normatively (e.g., morally or pragmatically) inapt, but it goes beyond the scope of this paper to identify the conditions under which this is the case.^14^ Furthermore, to say that what is felt over non-death losses during the pandemic is grief is not to commit to any claim about the felt severity of such grief–for example, how intense and long-lasting the suffering it involves is, or how difficult it is to accommodate the relevant losses. Thus, it is not to say that this ‘pandemic grief’ is as severe as bereavement grief any more than to say that a paper cut and childbirth both hurt is to commit to them hurting equally badly. Hence, since the claim that these are both pains does not trivialise the suffering of childbirth, likewise our claim does not trivialise bereavement grief.^15^ That said, whilst we are not committed to the felt severity of non-bereavement grief being equal to that of bereavement grief, it might also be a mistake to think that the former is necessarily always less severe than the latter. It is conceivable that *some* bereavement grief is less painful than the grief that is felt by someone who loses a job that is central to their sense of identity and their family’s only source of income, or their beloved family home, or even their sense of normalcy or safety during the pandemic.

In any case, there is a second way to reply to the trivialisation worry that bypasses these concerns that grief over cancellations and closures is not normatively appropriate. This line of response emphasises the fact that emotions directed at such happenings can form part of a grief process with a more encompassing object. It is widely accepted that grief is unlike simple short-lived emotions, like a brief episode of sadness or fear, in that it is instead a temporally extended *process* (e.g., Klass et al., 1996; Goldie, 2011; Ratcliffe, 2017). This process unfolds over time and allows for long temporal gaps where grief is not actively experienced. Grief’s process may include many diverse ingredients. As Goldie puts it, this process ‘includes characteristic thoughts, judgments, feelings, imaginings, actions, expressive actions, habitual actions, and much else besides […], but none of which is essential at any particular time’ (2011, p. 125). As such, grief understood as a process may be directed towards an overarching object, such as a significant loss or a loss of life possibilities, but also involve many constituent emotions (such as sadness, anger and disappointment) and other mental items directed at other objects, including subsidiary losses. As Ratcliffe (2017, p. 162) highlights, there is a kind of ‘two-sidedness’ to grief’s intentionality: it involves both ‘specifically directed intentional states’ but ‘these same experiences also have a more encompassing, diffuse structure’. This structure in the case of bereavement is, he suggests, due to the fact that the deceased may remain a condition of intelligibility for the experiential world, as discussed in the previous section. And grief’s process, in the case of bereavement, involves gradually recognising all of the ways that this experiential world depends (or depended) upon the deceased. Sometimes, this might involve recognising losses that, by themselves, may seem insubstantial—such as when giving away some old shirts to a charity shop or realising that there is nobody to tell about something funny that happened that day—but which in their wider context form constituents of grief.

This allows us to recognise that losses such as closures and cancellations during the pandemic may well be ‘objects’ of grief, even when they are not themselves elevated to the status of a significant loss. Someone’s overarching sense of loss during the pandemic may involve the gradual comprehension of many subsidiary–and perhaps taken individually–minor, losses, including cancellations and closures. Much like the case of bereavement, for some of those who experienced non-bereavement grief during the pandemic, the experience may not have been just of specific and concrete losses, but a disruption to the experience of the world. It is within the context of this profound disruption that cancellations of events and other seemingly trivial losses have been experienced, and these small losses may thereby be targets of grief.

One challenge that arises here is how we are to make sense of the idea of an ‘overarching’ grief process in the context of pandemic loss. This is a question about how, when a ‘minor’ loss is a subsidiary loss in an overarching ‘pandemic grief’ process, all the constituents of this process ‘hang together’. In virtue of what do these multifarious ingredients constitute aspects of an overarching experience of grief? In the case of bereavement grief, plausible answers can be given based upon the ways that different aspects of the emotional experience all relate to the person who died, one’s relationship with them (e.g., Cholbi, 2021),^16^ or how this person is woven into one’s experiential world (e.g., Ratcliffe, 2017). In such a case, it might be said that the disruption of grief is ‘as singular as the person who has died’ (Ratcliffe, 2017, p. 166). However, in the pandemic case, there isn’t a deceased person, relationship, or a singular lost object, in virtue of which the experience is unified.

As an initial response to this challenge, we can say that the different emotional constituents of the pandemic experience have at least a very loosely defined *common cause*: the Covid-19 pandemic. Some (Goldie, 2011; Ratcliffe, 2017) have argued that causal accounts alone do not suffice to explain the unified nature of grief,^17^ but we need not take it to be *solely* a common cause that determines how grief’s ingredients are tied together. We need not demand, for example, that *all* of one’s emotional experiences triggered by the pandemic (e.g., annoyance at the government’s ever-changing guidance or relief at being able to avoid a particular social gathering) are aspects of someone’s pandemic-related grief process.

Firstly, given an account of grief’s object, we can specify that only emotional experiences involving at least partial recognition of such an object can count. For example, on accounts where grief is directed towards losses of that in which one’s practical identity is invested (e.g., Cholbi, 2021) or losses of significant life possibilities (e.g., Ratcliffe, 2022), only experiences that are disclosive of such a loss, or the implications of such a loss, should count as part of the grief process. Feeling sad about an event cancellation is unlikely to involve recognition of the entirety of what has been lost during the pandemic, but where it forms part of a genuine grief process, it still discloses at least some aspect of this more overarching significant loss. In contrast, one’s relief about not having to attend a social event may not involve recognition of any aspect of such a loss, and thus it is not an aspect of a unified grief process.

Secondly, some of the constraints identified by those objecting to purely causal accounts of the unity of grief can be met in the non-bereavement case. For instance, Ratcliffe’s (2017) objection to such accounts is that grief involves relationships of *implication* rather than just contingent causal relationships. Certain kinds of experience are implied by prior experiences, insofar as they are to count as constituents of grief. For example,

Grief does not involve instantaneous, complete recognition of loss. But starting to grieve implies at least something about what is to come. A scenario where one ceases to grieve after 15 minutes, returns to a fully intact world, and continues to acknowledge the death is incoherent. (Ratcliffe, 2017, p. 167)

Grief, understood as a process of comprehension, cannot occur instantaneously. Although the components of non-bereavement grief lack certain relationships of implication that relate to the absence of a specific person, we can certainly allow that pandemic grief necessitates a more or less gradual process of ‘sinking in’ as the full implications of one’s losses are comprehended (see also Ratcliffe et al., 2022). Much like the bereavement case, to count as grief, the experience cannot only last 15 minutes, thereby suggesting that certain relationships of implication hold. Just as in the bereavement case, *starting* to grieve implies something about the continuation of a process of comprehension.

Moreover, much as the varied subsidiary losses of bereavement are experienced as tied together as aspects of a grief process, the losses of the pandemic are likewise generally not experienced in an atomistic manner. For example, the cancellation of a particular event may be experienced as closely tied to other cancellations and closures, which may relate to lost opportunities to meet people or to progress one’s career, which may relate to losses of community or security, and this may all add up to a very significant and disruptive sense of loss. Thus, treating cancellations and closures as merely isolated and trivial losses is a mistake; doing so may amount to dismissing each ingredient of a highly significant loss, one by one, such that we lose sight of the whole.

It might still seem, however, that responses to pandemic losses retain an element of vagueness in terms of how their constituents could hang together as grief. Although people might experience losses like cancellations and closures as being, to some degree, related to one another, this still contrasts with bereavement grief whose ingredients are tied together through their connection to the loss of a specific person. The emotional constituents of non-bereavement pandemic grief are generally not clustered around a singular significant concrete loss and so appear not to hang together in quite the way that the elements of a bereavement-grief process do.

In response, let us return to the notion, introduced in Section 4 of *ambiguous grief.* Paradigm instances of ambiguous grief over personal loss are of two kinds. *Psychological presence but physical absenc*e, as when a loved one goes missing (Boss, 1999, p. 8), and *physical presence but psychological absence*, as when a loved one is still alive but has cognitively declined (in dementia, for example) (Boss, 1999, p. 9). A pandemic grief process, where its object could be understood as (roughly) a diffuse and overarching loss not held together by a particular concrete loss, does not of course fit in either category since it does not involve a personal loss. Nevertheless, we can understand it as an experience of ambiguous loss in that similarly to these paradigm cases it can be hard to pinpoint what exactly is lost. The ambiguity involved in the pandemic grief process, whilst making it perhaps dissimilar to bereavement grief, does not preclude it from having sufficient unity to count as a grief process. Furthermore, the source of ambiguity in some pandemic-related non-bereavement grief is, in part, the fact that the disruption to one’s assumptive world is not unified as it may be in bereavement grief. If this is right, the losses of the pandemic plausibly need only be tied together in a loose and vague way in order to count as aspects of the same grief process. This vagueness maps onto the ambiguous phenomenology of the experience.

## Conclusion

We have defended the claim that we can take reports of grief over a range of non-death losses during the pandemic literally and as true. In particular, we can allow that such losses were grieved when–as in some first-person testimonies–they were not experienced as impermanent and also when the experience overlapped, phenomenologically, with the experience of bereavement grief. Both grief following bereavement and grief over the non-death losses of the pandemic involves disruption to the experiential world and to the socio-cultural scaffolding available to regulate one’s affective experiences. Whilst some reports of grief over minor losses might be best not taken literally, or as mistaken, we have also shown how such reports can be thought of as describing grief given an adequate understanding of the grief process. Such losses, we have suggested, can be the target of constituents of a grief process, the object of which might be thought of as a more overarching loss, such as the loss of a set of life possibilities. In the case of some pandemic non-bereavement grief, this set might be held together only loosely, yielding a kind of ambiguity.

## Footnotes

1 This is the title of Pinkser, 2020

2 Quotations from this study are from the dataset available at https://figshare.com/articles/dataset/_/15060138.

3 The full question was ‘As the COVID-19 pandemic continues, is there anything that you are grieving, mourning the loss of, or sad about? This may be concrete things, such as losing a loved one, job, community/family role, or daily routine; or more ambiguous, such as losing a sense of control, stillness at home, or belief that we can protect our family members. Please describe below’ (Statz et al., 2022).

4 The relevance for our purposes of how and whether speakers use the term grief or loss should not be overstated. It is possible for a subject to grieve but not know or believe that they are grieving, and thus not describe their experience in that way or intend such a description to be taken literally.

5 One exception is Dan Moller, who insists that ‘the proper object of grief is in fact the loss of the person’ (2017). However, he insists on this only in contrast to the idea that the grief following a bereavement takes as its object the ‘social and other roles that the deceased play in our lives’, and does not consider other candidate objects.

6 However, he also suggests (but does not defend the claim) that bereavement grief ‘is probably distinct from other traumas in both degree and kind’ (2021, p. 190).

7 These can in principle come apart. A subject may fail to grieve despite the presence of an appropriate worldly object or they may grieve inappropriately, in the absence of such an object. For the most part, this distinction is unimportant for our purposes here.

8 See also Ratcliffe 2017, 2019, 2020 on this account of grief’s object, and Ratcliffe 2022, pp. 154-7 for further discussion of non-death losses specifically.

9 For example, both Robert Neimeyer’s (e.g., 2005) view of grief as a process of ‘meaning reconstruction’ and Thomas Attig’s (2011) conception of grief as ‘relearning the world’ can be understood in this way.

10 The term ‘anticipatory grief’ was coined by Erich Lindemann (1944, p. 147) to describe the experience of grieving for someone before they are known to have died. The name ‘preparatory grief’ has been given to the anticipatory grief felt by a dying person. (Moon, 2016, p. 417) Where non-death losses are concerned, the distinction between ‘anticipatory’ and preparatory’ is not easily to be made.

11 Such losses have important implications for bodily processes, with bereavement loss experiences being compared to infant separation responses (Hofer, 1984, 1994) and drug withdrawal (Barrett, 2018, pp. 271–272) in terms of how they disrupt the body’s ability to retain allostatic balance (see also Atzil & Barrett, 2017).

12 Academic work has also emphasised the ways isolation can impact our bodies and our ability to experience the world as meaningful, highlighting another way in which the pandemic may cause disruption to one’s experience of the world. According to Lisa Guenther’s exploration of solitary confinement in prisons (2013), ‘Our overlapping, intercorporeal experience of other animate bodies, both human and nonhuman, sustains our own capacity to perceive the world as a meaningful context for our own lives’ (p. 155).

13 Thank you to a reviewer for this suggestion.

14 It might be argued that a distinctive feature of bereavement grief is that we have a duty to grieve such losses, and no others. Solomon (2004) argues that grief is a moral emotion in involving such a duty (see also McCracken, 2005, and Cholbi, 2021, who argues that we have if not a duty then a strong moral reason to grieve). In contrast, Richardson et al. (2021) suggest that whether or not we feel that someone ought to grieve is sensitive to the details of the loss incurred.

15 Although there may be circumstances in which the implication of stating such a comparison is one of equal severity. (‘It hurts!’ ‘Well, paper cuts hurt too.’)

16 Cholbi (2021) offers an account according to which grief is unified by virtue of being a species of emotional attention, all elements of which are directed towards different aspects of one’s relationship with the deceased.

17 Goldie (2011) argues that causal accounts fail to capture the ‘ironic’ blending of perspectives involved in the style of remembering central to grief’s process and argues that narrative explanations of grief do a better job in this regard. We do not discuss this idea further here, and remain neutral about the involvement of narratives in the grief process, but for critical discussion see Ratcliffe (2017).
